# Intracellular and Extracellular Expression of Bacillus thuringiensis Crystal Protein Cry5B in Lactococcus lactis for Use as an Anthelminthic

**DOI:** 10.1128/AEM.02365-15

**Published:** 2016-02-05

**Authors:** Evelyn Durmaz, Yan Hu, Raffi V. Aroian, Todd R. Klaenhammer

**Affiliations:** a Department of Food, Bioprocessing and Nutrition Sciences, North Carolina State University, Raleigh, North Carolina, USA; b University of Massachusetts Medical School, Worcester, Massachusetts, USA; Pennsylvania State University

## Abstract

The Bacillus thuringiensis crystal (Cry) protein Cry5B (140 kDa) and a truncated version of the protein, tCry5B (79 kDa), are lethal to nematodes. Genes encoding the two proteins were separately cloned into a high-copy-number vector with a strong constitutive promoter (pTRK593) in Lactococcus lactis for potential oral delivery against parasitic nematode infections. Western blots using a Cry5B-specific antibody revealed that constitutively expressed Cry5B and tCry5B were present in both cells and supernatants. To increase production, *cry5B* was cloned into the high-copy-number plasmid pMSP3535H3, carrying a nisin-inducible promoter. Immunoblotting revealed that 3 h after nisin induction, intracellular Cry5B was strongly induced at 200 ng/ml nisin, without adversely affecting cell viability or cell membrane integrity. Both Cry5B genes were also cloned into plasmid pTRK1061, carrying a promoter and encoding a transcriptional activator that invoke low-level expression of prophage holin and lysin genes in Lactococcus lysogens, resulting in a leaky phenotype. Cry5B and tCry5B were actively expressed in the lysogenic strain L. lactis KP1 and released into cell supernatants without affecting culture growth. Lactate dehydrogenase (LDH) assays indicated that Cry5B, but not LDH, leaked from the bacteria. Lastly, using intracellular lysates from L. lactis cultures expressing both Cry5B and tCry5B, *in vivo* challenges of Caenorhabditis elegans worms demonstrated that the Cry proteins were biologically active. Taken together, these results indicate that active Cry5B proteins can be expressed intracellularly in and released extracellularly from L. lactis, showing potential for future use as an anthelminthic that could be delivered orally in a food-grade microbe.

## INTRODUCTION

Intestinal roundworm (nematode) parasites, including hookworms, whipworms, and Ascaris, infect over a billion people worldwide and negatively affect growth, nutrition, cognition, and pregnancy (1). The World Health Organization has approved four anthelminthic drugs to treat roundworm infections: two benzimidazoles and two nicotinic acetylcholine receptor agonists (2). Resistance to these drugs is already widespread, leading to an urgent need for the development of new anthelminthics (2), and efforts are under way to develop Bacillus thuringiensis (Bt) crystal (Cry) proteins to treat intestinal nematode infections in humans. B. thuringiensis is a Gram-positive, spore-forming bacterium that produces parasporal crystalline protein inclusions known as Cry proteins (3). The Cry proteins are pore-forming proteins that bind to receptors on the intestines of invertebrates, causing impairment or death. A number of Cry proteins have been found to provide effective treatments against nematodes, and more specifically against parasitic helminths, *in vivo* (4, 5). Cry proteins have been shown to produce lethargy, anorexia, pale coloration, brood size reduction, developmental arrest, and/or death of roundworms. Cry proteins are proven to be safe for human consumption after over 50 years of use as crop insecticides, in aerial spraying, and incorporated into transgenic food crops, including organic crops (3).

One Bt Cry protein, Cry5B, has been shown to significantly reduce parasite burdens of (i) mice infected with the natural intestinal parasite Heligmosomoides polygyrus bakeri, (ii) hamsters infected with the zoonotic hookworm parasite Ancylostoma ceylanicum, and (iii) pigs infected with Ascaris (6, 7). Cry5B is a three-domain Cry protein similar in structure to the Cry1 family used in transgenic crops (3, 8). The genetic receptors for Cry5B in the nonparasitic roundworm Caenorhabditis elegans are carbohydrate structures present on lipids found on the roundworm intestinal surface, and these structures are absent in vertebrates (8). After binding of Cry5B to the receptors, protein monomers are proteolytically processed, oligomerize, and form pores in the plasma membrane of the intestine, causing death or severe damage to the intestinal surface of the nematode (8). A truncated version of Cry5B was cloned for expression in plant tissues and found to be highly toxic to C. elegans (9). The C-terminally truncated version retains the first 2,094 nucleotides of *cry5B* and the conserved five-amino-acid motif DRIEF, known as block 5, which occurs at the end of the active toxin domain of the Cry proteins (9, 10).

The food-grade bacterium Lactococcus lactis has been used to express a variety of vaccines and biotherapeutics (11–13). L. lactis does not colonize mammalian digestive tracts (14). Colonization is not a desirable feature for a bacterial protein delivery system to the gastrointestinal tract (GIT). Ideally, the live delivery bacteria would be administered, survive for some time, and then be washed through and out of the GIT. L. lactis is known to be acid and bile sensitive and is thus vulnerable in the mammalian stomach and small intestine (15). However, this susceptibility is strain dependent and can be altered if L. lactis is protected with milk or food, either *in vitro* or *in vivo* (15–17). L. lactis can be incorporated into a food matrix or a fermented milk product or can be freeze-dried and encapsulated (18, 19). In the GIT, Cry5B-containing L. lactis would release the protein upon lysis (15, 16). Alternatively, Cry5B could be exported from surviving L. lactis cells into the GIT, or killed L. lactis cells could be administered as an abiotic (20). Interestingly, viable L. lactis is metabolically active in each compartment of the rat GIT, while dead cells are rapidly lysed (16). Recent studies have shown that even dead bacteria administered as probiotics can have significant effects on the host, particularly on the immune system (20, 21). Thus, either live or dead, L. lactis cells could function as a safe delivery vehicle for Cry5B, which would eventually be released directly into the intestinal environment of the helminth population.

Here we test the hypothesis that such a food-grade bacterium could be engineered to express and release full-length Cry5B and its truncated form (tCry5B), potentially to target nematode parasites in the GIT. This study presents two systems in Lactococcus lactis developed for the successful intracellular expression and externalization of full-length and truncated Cry5B.

## MATERIALS AND METHODS

### Bacterial strains and culture conditions.

Strains and plasmids used in this work are shown in Table 1. L. lactis MG1363 and KP1 were propagated aerobically without shaking at 30°C in Difco M17 medium (Becton, Dickinson and Company, Sparks, MD) supplemented with 5% glucose (M17G). Escherichia coli DH5α (Life Technologies, Grand Island, NY) and XLI-Blue (Agilent Technologies, Inc., Santa Clara, CA) were propagated with shaking in Luria-Bertani broth (LB) or on Bacto brain heart infusion (BHI) agar plates at 37°C (Becton Dickinson). Cultures were stored at −20°C in the appropriate medium supplemented with 10% glycerol. As necessary, growth media were supplemented with 1.5 or 150 μg/ml erythromycin (Em) for L. lactis or E. coli, respectively. Experiments for growth curves were performed in test tubes by using a Bausch and Lomb Spectronic 20 or in 96-well plates by using a FLUOstar Optima plate reader (BMG Labtech, Inc., Durham, NC).

**TABLE 1:** Bacterial strains and plasmids used in this work

Bacterial strain or plasmid	Description	Source or reference(s)
Strains		
Escherichia coli DH5α	Cloning host	Invitrogen
Escherichia coli XL1-Blue	Cloning host	Stratagene
Lactococcus lactis MG1363	Expression host; plasmid cured	41, 44
Lactococcus lactis KP1 (NCK203)	Expression host; 2 native plasmids and 2 prophages	45
Lactococcus lactis subsp. lactis ATCC 11454	Nisin producer and nisin-resistant L. lactis strain	ATCC^*a*^
Plasmids		
pQE9 Cry5B	Source of *cry5B* gene for cloning	46
pSC-B	PCR cloning vector	Agilent Technologies
pTRK593	pTRKH2-based cloning vector with P6 promoter^*b*^	23
pTRK1040	Bt Cry protein Cry5B cloned into pTRK593	This study
pTRK1041	Bt Cry protein tCry5B cloned into pTRK593	This study
pTRK1061	pTRKH2-based expression vector with leaky system promoter and activator	This study
pTRK1062	Bt Cry protein Cry5B cloned into pTRK1061	This study
pTRK1063	Bt Cry protein tCry5B cloned into pTRK1061	This study
pMSP3535H3	Nisin expression vector with nisin and erythromycin resistance genes	34
pTRK1068	Bt Cry protein Cry5B cloned into pMSP3535H3	This study
pTRK1069	Bt Cry protein tCry5B cloned into pMSP3535H3	This study

a American Type Culture Collection, Manassas, VA.

b pTRKH2 is a 6.9-kb high-copy-number E. coli/Gram-positive shuttle vector encoding erythromycin resistance (32).

### Plasmid construction.

Primers for PCR cloning were obtained from Integrated DNA Technologies (Coralville, IA). PCR was accomplished using the *Pfu* Ultra II Fusion HS DNA polymerase (Agilent Technologies, Inc.). Restriction enzymes were obtained from Roche Applied Science (Indianapolis, IN). DNA fragments were gel purified using a QIAquick gel extraction kit, and PCR products were purified using a QIAquick PCR purification kit (Qiagen Sciences, Valencia, CA). DNA ligations were carried out with a Fast-Link DNA ligation kit (Epicentre Biotechnologies, Madison, WI). The ligation mixes were transformed into E. coli XL1-Blue or DH5α by using a Z-Competent E. coli transformation buffer set (Zymo Research, Orange, CA). Transformation isolates were selected on BHI agar plates with 150 μg/ml Em and propagated in LB with 125 μg/ml Em. Plasmid DNA was isolated using a QIAprep Spin miniprep kit. Cloned isolates were confirmed by sequencing at Eton Biosciences (Research Triangle Park, NC) or Davis Sequencing (Davis, CA), directly from plasmid DNA or from PCR products obtained using Choice *Taq* Blue DNA polymerase (Denville Scientific, Metuchen, NJ). Plasmids were visualized *in silico*, and DNA sequences were aligned using the Clone Manager 9, professional edition (Scientific and Educational Software, Cary, NC), and Geneious Pro (Biomatters Ltd., Auckland, NZ) software packages. Confirmed plasmids were electroporated into L. lactis as described previously (22). Plasmids were isolated from L. lactis transformants by use of QIAprep Spin miniprep kits following the manufacturer's instructions, but with an added 15 min of incubation in buffer P1 at 37°C with 5 to 6 mg lysozyme (Sigma-Aldrich Co., St. Louis, MO) prior to the addition of buffer P2. The L. lactis clones were confirmed by sequencing of PCR products amplified from the plasmid DNA by using the M13 F and M13 R primers.

The full-length (3,792 bp) and truncated (2,126 bp) versions of the *cry5B* gene were initially cloned into pTRK593 downstream of the constitutive P6 promoter (23). PCR products were amplified from pQE9-Cry5B by use of primers encoding an upstream SalI site (italics), a ribosome binding site (underlined), and a start codon (forward primer Cry5B-RBS-SalI [GATC*GTCGAC*AAGGAGAACGTATATGGCAACAATTAATGAGTTGTATC]), as well as downstream PstI sites (italics) (reverse primer for full-length Cry5B, pQE9-R-PstI [GATC*CTGCAG*TATCCAAGCTCAGCTA]; and reverse primer for truncated Cry5B, pQE9-R-tCry5B [GATC*CTGCAG*ATCAGTCTATTGGATT]). The truncated version of Cry5B ends two amino acid residues after the box 5 feature of Cry5B, with the relevant coding sequence followed by a stop codon (9). The Cry5B PCR products were cloned into Stratagene plasmid pSC-B via a StrataClone Blunt PCR cloning kit following the manufacturer's instructions (Agilent Technologies, Inc., Santa Clara, CA), and from there were cloned into pTRK593 (23). The *cry5B* insert DNA was removed from pSC-B by use of PstI, SalI, and BglI. BglI was used to digest the pSC-B vector into smaller pieces, since it is almost the same size as the *cry5B* fragment. The truncated *cry5B* insert was removed with PstI and SalI. Each insert was ligated into PstI/SalI-digested pTRK593 DNA.

Cloning for Cry5B expression by the leaky Lactococcus system was accomplished using vector pTRK1061. The vector was constructed from pTRK617 (24) by using an XhoI/SalI double digest and religation, removing the tetanus toxin fragment C (TTFC) gene. The following primers were used to amplify *cry5B* from pTRK1040: forward, Cry5B RBS PstI (GATC*CTGCAG*AAGGAGAACGTATATGGCAACAATTAATGAGTTGTATC); and reverse, Cry5B XhoI R (GATC*CTCGAG*TATCCAAGCTCAGCTAATTAAG). The reverse primer for truncated Cry5B was tCry5B XhoI R (GATC*CTCGAG*ATCAGTCTATTGGATTTTTGGAAC). The Cry5B fragments and vector pTRK1061 were digested with PstI and XhoI and ligated.

For nisin-induced expression of Cry5B by use of vector pMSP3535H3, full-length and truncated *cry5B* genes were PCR amplified from pTRK1040 by using the following primers: cry5B SphI forward (GATC*GCATGC*GTGAGGAGAACGTATATGGCAACAATTAATGAGTTG) and cry5B BamHI reverse (GATC*GGATCC*GCAGTATCCAAGCTCAGCTAATTAAG). Truncated Cry5B was amplified with truncated cry5B BamHI reverse (GATC*GGATCC*GCAGTATCCAAGCTCAGCTAATTAAG). The PCR products were cloned into vector pSC-B by using a StrataClone Blunt PCR cloning kit, and from there into pMSP3535H3 by using the SphI and BamHI restriction sites encoded in the PCR primers.

### Immunoblotting.

L. lactis cultures were propagated for 16 h and then transferred from overnight cultures and propagated in 10 ml M17G with Em (1.5 μg/ml) to an OD_600_ of 0.5 to 0.6 (log phase), followed by centrifugation to pellet cells. Supernatants and pellets were frozen separately at −80°C for storage. Cell supernatants were filtered through 30,000-molecular-weight-cutoff Amicon Ultra centrifugal filters following the manufacturer's instructions (Millipore Ireland Ltd., Tullagreen, Ireland). The filters were then washed with 4 ml of phosphate-buffered saline (PBS), pH 7.4 (Invitrogen, Grand Island, NY), and the concentrated proteins were eluted with 50 μl of PBS. Cell pellets were resuspended in 1 ml PBS and then homogenized with a Mini BeadBeater 8, using 0.1-mm glass beads in 2-ml screw-cap tubes, for three 1-min intervals interspersed with 1 min on ice to produce cell lysates (Biospec Products, Bartlesville, OK). Protein concentrations in all samples were determined using the Bradford reagent (Sigma-Aldrich Co., St. Louis, MO), and approximately 7 to 10 μg protein per lane was electrophoresed in Mini-Protein 7.5% or 4.5 to 10% TGX gels (Bio-Rad, Hercules, CA) and then transferred to polyvinylidene difluoride (PVDF) membranes by use of a Bio-Rad Trans-Blot Turbo transfer system following the manufacturer's instructions. The membranes were hybridized with Cry5B primary antibodies (Thermo Fisher Scientific, Waltham, MA) and with goat anti-rabbit IgG(H+L)–horseradish peroxidase (HRP) conjugate (Bio-Rad) and developed with Pierce ECL Western blotting substrate (Fisher Scientific, Pittsburgh, PA) or Clarity Western ECL substrate (Bio-Rad) following the manufacturer's instructions.

### Nisin induction.

Stock solutions of 1 mg/ml nisin in 0.02 N HCl were prepared from a preparation of 2.5% nisin obtained from Sigma-Aldrich Co. (St. Louis, MO). Ten-milliliter cultures were grown to an optical density at 600 nm (OD_600_) of 0.3 to 0.4 in M17G with Em after inoculation from 18-h cultures. Nisin was added at various dilutions to the cultures, and incubation was continued for 3 h before harvesting cells and supernatants for comparison of protein expression levels by Western blotting. For growth curve experiments, control strains were grown to an OD_600_ of 0.4 and then incubated for an additional 3 h without the addition of nisin (the final OD_600_ was 1.0 to 1.1).

### LDH activity assays.

Lactate dehydrogenase (LDH) activity was detected indirectly by measuring the oxidation of NADH in the presence of pyruvate and fructose 1,6-diphosphate (FDP) at 340 nm (25, 26). The protocol was modified for use with a well mode assay in a FLUOstar Optima plate reader. Supernatant samples were removed from centrifuged 1-ml log-phase cultures (OD_600_ = 0.5) and transferred to a separate tube. A reagent mix of Tris, pH 6.8 (0.05 M), sodium pyruvate (0.1 M), and FDP (30 mM) (all chemicals were from Sigma-Aldrich) was aliquoted at 223 μl per well into the wells of a 96-well plate. Samples (20 μl) of cell supernatants were added to the wells in triplicate. NADH (25 μl of 1.5 μM NADH in Tris buffer, pH 8.5) was separately injected into each well, followed by 2 s of shaking and five consecutive OD_340_ readings spaced 30 s apart. The endpoints of the reactions were observed, and LDH activity was calculated from the slope of the kinetic curve for each well.

### LIVE/DEAD assays.

To assay cell permeability, a LIVE/DEAD BacLight bacterial viability kit (Life Technologies, Grand Island, NY) was used according to the manufacturer's instructions. The kit uses two nucleic acid-binding stains to differentiate cells with intact membranes (live cells) from those with compromised membranes (dead cells). SYTO 9 is a green, cell-permeant dye that labels all bacteria, regardless of membrane integrity. Propidium iodide (PI) is a red dye which labels cells with compromised or damaged cell membranes. A standard curve was prepared according to the manufacturer's instructions, using standard ratios of untreated log-phase cells and log-phase cells treated with 70% isopropyl alcohol for 1 h. For the assay, 6 to 10 ml of untreated or treated log-phase cells was centrifuged, resuspended in 4 ml of 0.85% NaCl, and adjusted to an OD_690_ of 0.3. One-hundred-microliter aliquots of adjusted cells were added in triplicate to the wells of a black 96-well plate (Nunc; Thermo Scientific, Waltham, MA) with 100 μl of an equal mixture of kit reagents A and B (3.34 mM SYTO 9 dye, with 480/500-nm excitation/emission wavelengths; and 20 mM propidium iodide, with 490/635-nm excitation/emission wavelengths), each diluted 3:2,000 (vol/vol) in H_2_O. After mixing, the plates were held for 15 min in the dark and then read at 490/530- and 490/600-nm excitation/emission settings on a FLUOstar Optima plate reader. Ratios of SYTO 9 signals to PI signals were determined, and the results were used to calculate the percentage of undamaged cells based on the standard curve.

### C. elegans assays.

A C. elegans *sek-1* (*km4*) strain was used in a bioactivity assay (27). SEK-1 is a mitogen-activated protein kinase kinase (MAPKK) that is immediately upstream of the C. elegans p38 MAPK, PMK-1, and immediately downstream of the MAPKK kinase, NSY-1 (28, 29). C. elegans sek-1 (*km4*) was maintained using standard techniques, with E. coli strain OP50 as a food source (30). Assays were performed at 25°C as previously described for developmental assays (31), except that in place of S medium, 200 μl of L. lactis MG1363 cell pellet lysate was added to each well. Cell lysates were prepared in PBS as described above. E. coli OP50 cells were propagated to an OD_600_ of 3.0, and 40 μl was added per well with 10 to 15 *sek-1* (*km4*) first-life-stage larvae and 0.2 μl 5-mg/ml cholesterol (in ethanol) as the food source. Assays were performed in 48-well plates, with two wells per condition, in each of three independent trials. Images of C. elegans were captured using an Olympus BX-60 microscope at 48 h of incubation. The images shown in Fig. 7 are representative of the results observed over the three trials.

## RESULTS

### Expression of Cry5B and tCry5B from pTRK593 for high-level constitutive expression.

Vector pTRK593 (23), which is based on the theta replicating plasmid pTRKH2 (32), replicates in E. coli and Lactococcus with a high copy number. The genes encoding the Bt Cry proteins Cry5B and tCry5B were cloned behind a strong, constitutive promoter, P6, positioned upstream of a multiple-cloning site in pTRK593. Plasmids pTRK1040 (pTRK593::cry5B) and pTRK1041 (pTRK593::tcry5B) were first obtained in E. coli and then transformed into L. lactis strains MG1363 and KP1.

**FIG 1:**
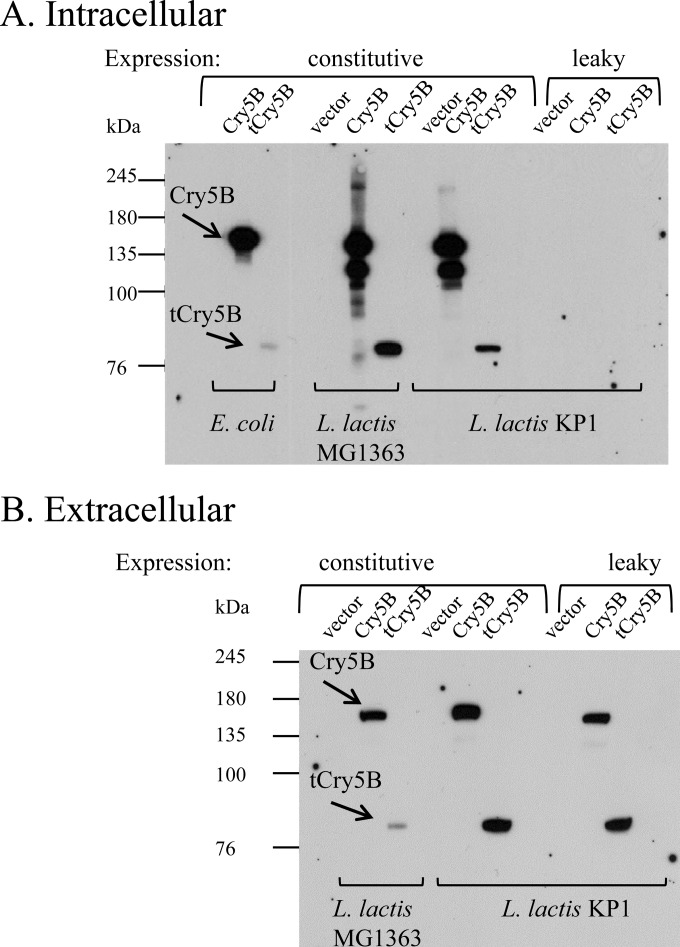
Immunoblots showing Cry5B and truncated Cry5B (tCry5B) expression, constitutively in log-phase cultures (vector, pTRK593; Cry5B, pTRK1040; and tCry5B, pTRK1041) of E. coli and L. lactis MG1363 and KP1 and leakily from a leaky vector (pTRK1061) in L. lactis KP1 (Cry5B, pTRK1062; and tCry5B, pTRK1063). (A) Cell lysates. (B) Cell-free supernatants.

Immunoblots confirmed the intracellular expression of the Bt Cry proteins in both Lactococcus backgrounds (Fig. 1A, “constitutive” lanes). Full-length Cry5B was partially degraded in the Lactococcus intracellular samples (Fig. 1A) but not in the extracellular samples (Fig. 1B). The mechanism of degradation is unknown, but degradation commonly occurs when heterologous proteins are expressed intracellularly in L. lactis (33). Significant proportions of the Cry5B and tCry5B proteins were also found in the cell supernatants of the log-phase cultures of both lactococcal expression hosts (Fig. 1B, “constitutive” lanes). The results clearly show the expression and release of Cry5B and truncated Cry5B for Lactococcus lactis MG1363 and KP1.

### Cry5B expression through nisin induction via a high-copy-number plasmid.

In attempts to increase expression in Lactococcus, the Cry5B and tCry5B inserts were cloned into plasmid pMSP3535H3 (34). Plasmid pMSP3535H3 is a derivative of the original nisin expression vector pMSP3535 with an improved promoter, a bidirectional terminator, and a replication region derived from pTRKH2. The *nisI* nisin resistance gene is also carried, which allows induction with higher levels of nisin than was possible with the original vector (32, 34, 35). The plasmids produced using this vector were pTRK1068 (pMSP3535H3::cry5B) and pTRK1069 (pMSP3535H3::tcry5B). These plasmids were transformed into L. lactis MG1363.

**FIG 2:**
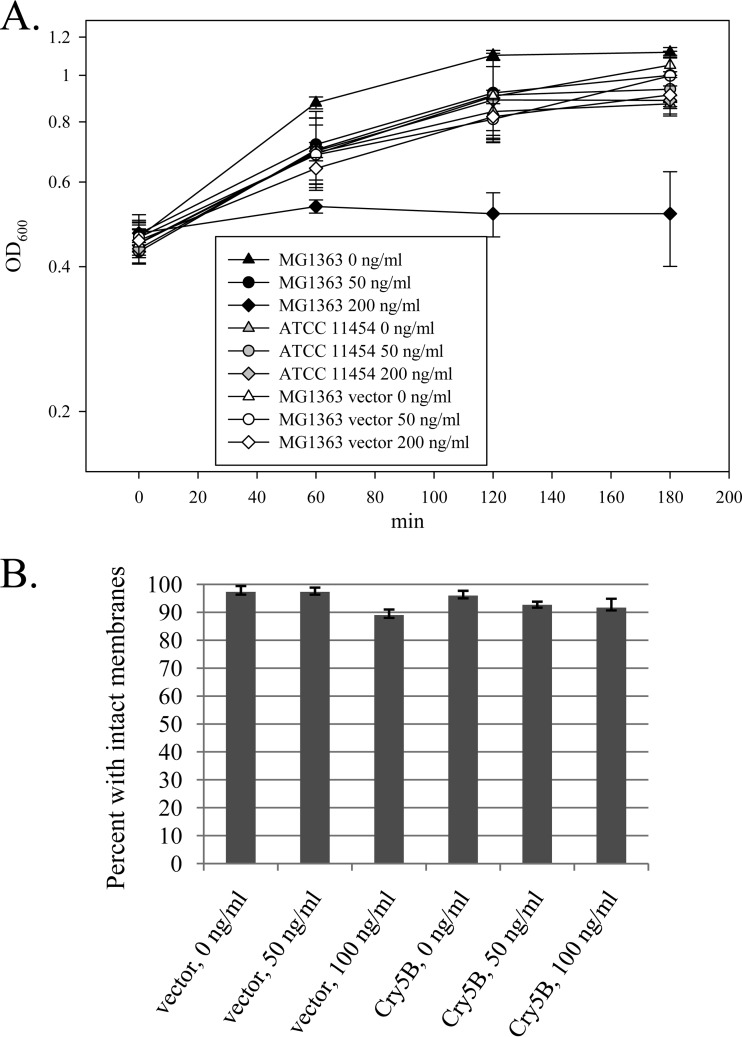
Effects of added nisin on L. lactis MG1363. (A) Growth curves after the addition of nisin to log-phase cultures at an OD_600_ of ∼0.4, showing data for the Lactococcus host MG1363 parent strain (nisin sensitive), MG1363 harboring the base nisin-inducible vector (pMSP3535H3), and Lactococcus lactis ATCC 11454, a nisin-producing, naturally nisin-resistant L. lactis strain. Averages for three independent experiments are shown. (B) LIVE/DEAD BacLight assay showing the effects of increasing nisin concentrations on the membrane permeability of L. lactis MG1363 cells transformed with either the nisin-inducible vector (pMSP3535H3) or the vector with the full-length Cry5B insert (pTRK1068).

The effects of added nisin on the L. lactis MG1363 cultures containing pMSP3535H3-based plasmids were investigated. Growth curves were generated using MG1363, MG1363 plus vector (pMSP3535H3), and L. lactis subsp. lactis ATCC 11454 (a nisin-producing strain which is naturally nisin resistant), with nisin added at 0, 50, and 200 ng/ml (Fig. 2A). The results show that the parent strain MG1363 was significantly inhibited by high levels of nisin, whereas MG1363 carrying the nisin expression vector grew uninhibited at high levels of nisin (up to 200 ng/ml), as did the naturally nisin-resistant, nisin-producing control strain, ATCC 11454. The cell membrane permeability of nisin-induced cells was tested using the LIVE/DEAD BacLight assay. This assay uses the following two fluorescent dyes: the green dye SYTO 9, which stains all cells; and the red dye propidium iodide (PI), which is taken up only into cells with a compromised membrane. The ratio of fluorescence values for the two dyes is used to calculate the percentage of “live” cells from a standard curve. Here we used the assay as an indication of increased membrane permeability, as reflected by a lower percentage of “live” cells (cells with an intact membrane). The percentage of cells with an intact membrane was only slightly affected by the addition of nisin to log-phase cells (Fig. 2B).

**FIG 3:**
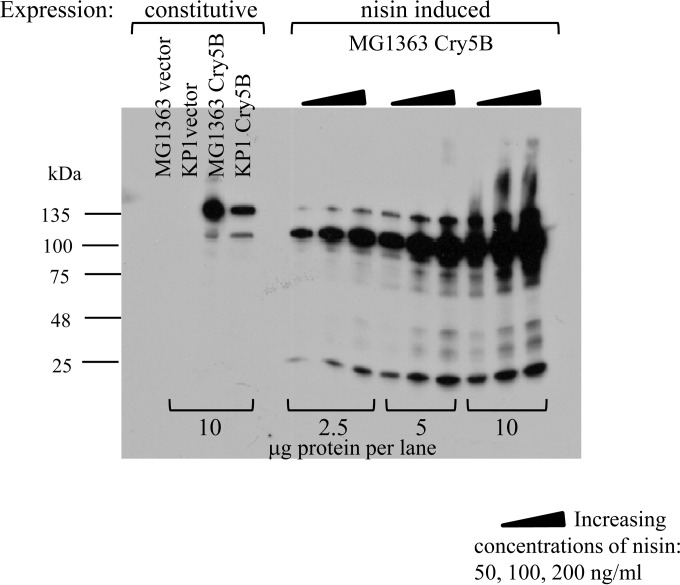
Nisin induction of Cry5B expression from Lactococcus lactis. Controls were L. lactis MG1363 and KP1 with vector (pTRK593) and with constitutive expression of Cry5B (pTRK1040). Nisin-induced expression of Cry5B was from MG1363 with pTRK1068, with increasing concentrations of nisin as indicated by triangles. Samples were loaded at different protein concentrations (2.5, 5, or 10 μg protein per lane).

Immunoblots were used to investigate the effects of various levels of nisin induction on Cry5B expression (Fig. 3). At 50, 100, and 200 ng/ml nisin induction, Cry5B demonstrated strong induction; however, the Cry5B protein appeared to be processed, with a reduction of its apparent size from 140 kDa to about 120 kDa. This processing was evident when Cry5B was expressed from the P6 promoter in pTRK1040 in both MG1363 and KP1 (lanes 3 and 4) but was more prominent after nisin induction (lanes 6 to 14). Cell supernatants were also tested; however, significant amounts of Cry5B were not detected (data not shown). Various amounts of cell lysate proteins were loaded into SDS-PAGE gels (2.5, 5, and 10 μg/lane), and immunoblotting was used to determine the relative amounts of Cry5B produced by nisin induction in MG1363 (nisin induced; pTRK1068) compared with the base level produced in either L. lactis MG1363 or KP1 harboring the constitutive expression plasmid pTRK1040 (Fig. 3). Comparing nisin-induced expression in MG1363(pTRK1068) (Fig. 3, lanes on the right [10 μg protein/lane]) with constitutive expression from MG1363(pTRK1040) (Fig. 3, left lanes [constitutive]) showed that a significantly larger amount of total Cry5B was induced with 200 ng/ml nisin than the level obtained with the base expression vector. However, comparable amounts of full-length (140 kDa) Cry5B were expressed from both pTRK1040 and pTRK1068 (Fig. 3, compare lanes 3 and 14).

### Externalized expression of Cry5B by use of the leaky Lactococcus system.

For use of the protein as an anthelminthic, we sought L. lactis strains that produced Cry5B intracellularly, and also strains capable of exporting or externalizing Cry5B. The leaky Lactococcus system (24) was investigated for possible externalization of the Cry5B protein. Previously, L. lactis KP1 (NCK203) was shown to externalize tetanus toxin fragment C (TTFC) expressed from pTRK617 (24). Plasmid pTRK617 contains an 888-bp fragment of the lytic lactococcal bacteriophage φ31, which carries a late phage promoter (P_15A10_) and encodes Tac31A, a phage transcriptional activator. The leaky phenotype depends on the presence in the lactococcal host strain of the plasmid-encoded activator as well as a resident prophage which carries a phage promoter homologous to P_15A10_. Low-level activation of the resident prophage holin and lysin gene cassette in *trans* from the plasmid produces the leaky phenotype (24). The leaky vector pTRK1061 was constructed through deletion of the TTFC gene of pTRK617. Plasmids pTRK1062 (pTRK1061::Cry5B) and pTRK1063 (pTRK1061::tCry5B) were produced and were transformed into L. lactis KP1, which contains at least two prophages and was used as the plasmid host strain to leak TTFC in previous work (24).

**FIG 4:**
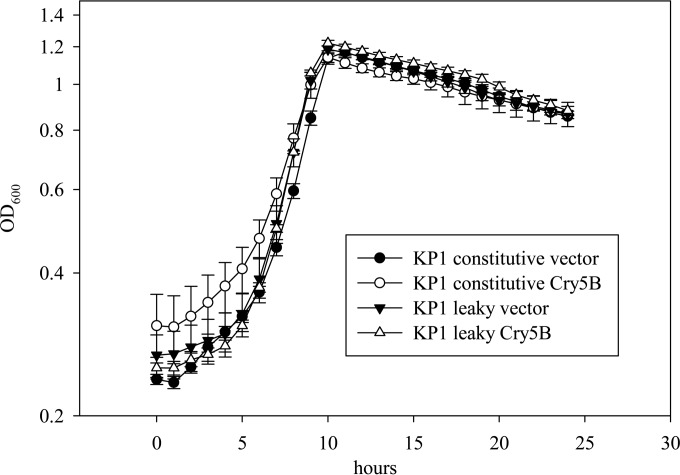
Growth curves for L. lactis KP1 with the base constitutive expression plasmid vector (pTRK593), the constitutive Cry5B expression vector (pTRK1040), the base leaky vector (pTRK1061), and the leaky Cry5B expression vector (pTRK1062).

Immunoblotting revealed that virtually all of the Cry5B and tCry5B produced in log-phase KP1 cells with the leaky system appeared in the cell supernatant (Fig. 1B, “leaky” lanes [pTRK1061 based]), with little to none remaining in the cell pellet lysates (Fig. 1A, “leaky” lanes [pTRK1061 based]). However, the amount of Cry5B or tCry5B in the supernatants was not markedly greater than that observed in the supernatants where Cry5B and tCry5B were expressed from the stronger P6 promoter of pTRK593. Similar to that in the previous experiments with TTFC externalization (24), cell growth was not affected (Fig. 4).

**FIG 5:**
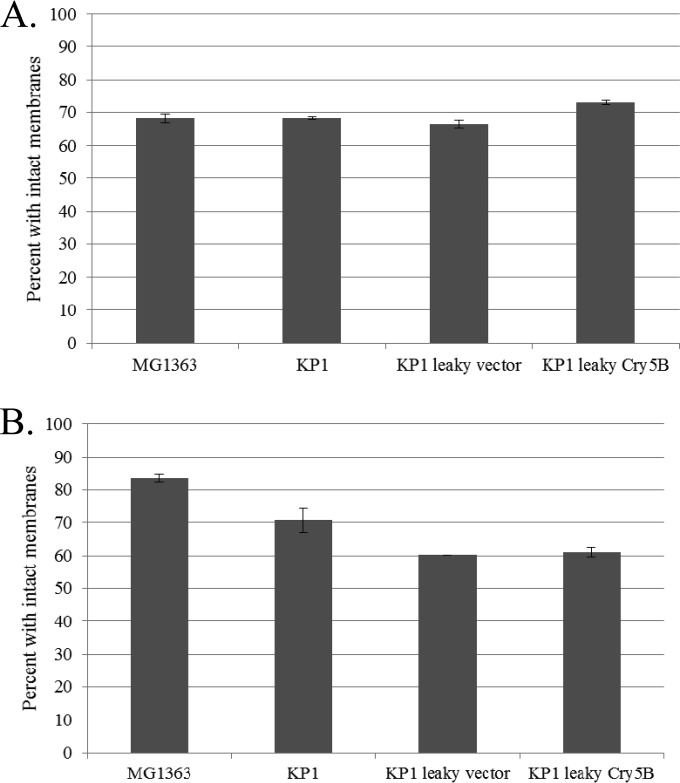
Results of representative LIVE/DEAD assays performed with a BacLight kit, showing the membrane permeabilities of log-phase cells (A) and overnight/stationary-phase L. lactis cells (B) of the following strains: MG1363, KP1, and KP1 with the leaky vector (pTRK1061) or leaky expression of Cry5B (pTRK1062).

Cell membrane permeability assays using a LIVE/DEAD BacLight kit were conducted to determine whether increased cell permeability contributed to the externalization of Cry5B in the leaky cells (Fig. 5). Increased permeability of leaky L. lactis KP1 log-phase cells compared to that of nonleaky cells or MG1363 cells was not detected (Fig. 5A). This result agrees with the previous study, where no increase in cell permeability was detected using propidium iodide with log-phase cells (24). However, when overnight cells were tested (Fig. 5B), distinct differences were observed. Overnight L. lactis MG1363 cells were less permeable than those in the log phase, whereas KP1 cells were unchanged. Among the overnight samples, KP1 was distinctly more permeable than MG1363, and KP1 carrying the leaky vector or the leaky vector expressing Cry5B was significantly more permeable than KP1 without a leaky plasmid. These results might be expected for stationary-phase cells exhibiting a holin/lysin-induced leaky phenotype.

**FIG 6:**
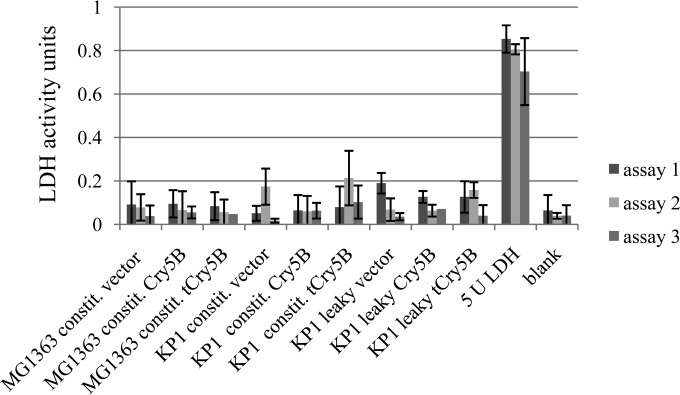
LDH enzyme activity assays of supernatants from log-phase L. lactis cells in three experiments. LDH (5 U) was used as a positive control. The constitutive (constit.) vector was pTRK593; the constitutive Cry5B and truncated Cry5B (tCry5B) strains contained pTRK1040 and pTRK1041, respectively; the leaky vector strains contained the vector plasmid pTRK1061; the leaky Cry5B strain contained pTRK1062; and the leaky truncated Cry5B (tCry5B) strain contained pTRK1063.

Lactate dehydrogenase (LDH; 35 kDa) assays were used to investigate whether or not Cry5B was released into the cell lysate through the normal process of cell lysis in a bacterial population. Normally, LDH is an intracellular enzyme and is not found in cell supernatants unless cell lysis occurs (36). LDH experiments repeated three times with log-phase samples of the leaky strains and controls were used to compare LDH activities in the cell supernatants among the leaky and nonleaky strains (Fig. 6). Extracellular LDH activity was negligible in all the supernatant samples. These data indicate that cell lysis was not occurring in the log-phase cells and that lysis was not responsible for the external release of the Cry proteins but likely occurred as a result of the phage activator Tac31A and its effect on the late holin and lysin genes of the resident KP1 prophage.

### Biological activity of Cry proteins.

**FIG 7:**
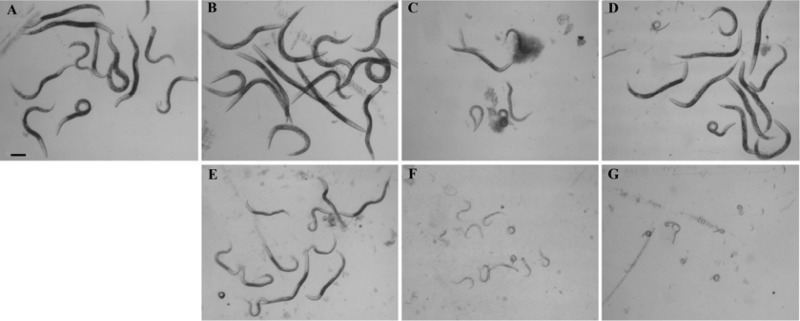
Biological activity assays of Cry5B proteins expressed from L. lactis. C. elegans *sek-1* (*km4*) worms were challenged with cell lysates of L. lactis MG1363 producing Cry5B or tCry5b from constitutive or nisin-inducible expression plasmids. (A) MG1363 host strain; (B) constitutive expression vector only (pTRK593); (C) constitutively expressed Cry5B from pTRK593 (pTRK1040); (D) constitutively expressed tCry5B from pTRK593 (pTRK1041); (E) base nisin-inducible plasmid (pMPS3535H3); (F) nisin-induced Cry5B from pMPS3535H3 (pTRK1068); (G) nisin-induced tCry5B from pMPS3535H3 (pTRK1069).

To assess the biological activity of Cry5B expressed in L. lactis, bioactivity assays of cell lysates of L. lactis MG1363 strains producing Cry5B and tCry5B were performed using a laboratory roundworm model, C. elegans *sek-1* (*km4*). *sek-1* (*km4*) animals carry a mutation in the MAPKK pathway which leads to hypersusceptibility of this C. elegans strain to Cry5B (27–29). Representative results of one of three independent trials are shown in Fig. 7. In Fig. 7A, *sek-1* (*km4*) worms treated with the host strain MG1363 show no effect of the added bacterial cell lysate. Similarly, lysates from MG1363 with the constitutive vector (pTRK593) or with constitutively expressed tCry5B (pTRK1041) did not show an effect (Fig. 7B and D). However, cell lysates of cells constitutively expressing Cry5B (pTRK1040) (Fig. 7C) were clearly intoxicating to the worms. Cell lysate from MG1363 containing the nisin-inducible empty vector (pMSP3535H3) also had an effect on the *sek-1* (*km4*) C. elegans worms, likely due to the worms' sensitivity to nisin, which was added to the culture of the L. lactis control strain prior to cell lysate preparation. However, there was a clear additional effect of both nisin-induced Cry5B (from pTRK1068) (Fig. 7F) and nisin-induced tCry5B (from pTRK1069) (Fig. 7G). These assays demonstrate a striking biological activity of both Cry5B and tCry5B in the *sek-1* (*km4*) C. elegans strain. In addition, these results suggest the presence of biological activity from the processed version of full-length Cry5B shown in the immunoblot in Fig. 3 (120 kDa) compared with unprocessed Cry5B (140 kDa), since most of the nisin-induced Cry5B from pTRK1068 is in the processed form.

## DISCUSSION

In this study, we demonstrated that the B. thuringiensis Cry protein Cry5B (140 kDa) and a truncated form, tCry5B (79 kDa), can be expressed intracellularly in and released extracellularly from L. lactis. This is the first step toward the use of L. lactis as a delivery vehicle for Cry5B in the treatment of mammalian helminth infections.

New anthelminthics must be inexpensive and produced in large quantities for delivery under adverse environmental conditions (4). Ideally, they will not require cold storage and can be delivered in a single dose. L. lactis is a food-safe, generally recognized as safe (GRAS) organism which can be engineered to deliver Cry proteins safely and economically for treatment of helminth infections.

In this study, Cry5B was overexpressed by using nisin induction. Nisin is an antimicrobial peptide secreted by various strains of L. lactis and can be used as a natural preservative. Nisin induction has been used widely in L. lactis and other bacteria for expression of heterologous proteins, and it is described as being simple to use, with high yields (11). The L. lactis nisin operon consists of 11 genes. Among the gene products, the NisA protein is the active peptide, and its production is controlled by NisR, a regulator protein, and NisK, a membrane kinase which is activated in the presence of nisin (37). NisI appears to act cooperatively with NisFEG to provide cellular immunity to nisin (37). The *nisI*, *nisR*, and *nisK* genes are present in pMSP3535H3, the expression vector used in this work. NisI provides enough added resistance to nisin to enable growth in the presence of up to 200 ng/ml nisin (34). Cry5B was strongly induced by nisin compared to the base level of expression from the strong constitutive promoter carried on pTRK593. However, most of the nisin-induced Cry5B protein was reduced in size from 139 kDa to about 120 kDa. The reason for this clipping is unknown. Nevertheless, biological assays with C. elegans sek-1 (*km4*) worms demonstrated that the nisin-induced Cry5B and tCry5B proteins effectively halted worm development.

Cytoplasmic proteins without signal sequences are commonly found in cell supernatants due to cell lysis. Intriguingly, a study of cytoplasmic proteins found in Staphylococcus aureus cell supernatants was done by comparing wild-type cells with mutants in the major autolysin Atl (38). It was found that the presence or absence of prophages had little effect on the secretome of S. aureus. Twenty-two cytoplasmic proteins which were present in the secretome of the wild type were significantly decreased in the *atl* mutant, confirming a role for the autolysin in release of the proteins. A selection mechanism for the excretion of cytoplasmic proteins was postulated but not characterized. Lactococcus lactis is known to exhibit extensive autolysis capability (39). It is possible that low-level expression of an autolysin enables externalization of Cry5B expressed constitutively from pTRK1040 and pTRK1041 in L. lactis.

The leaky Lactococcus system used here to externalize Cry5B and tCry5B depends on the constitutive activation of a prophage holin and lysin gene cassette by a transcriptional activator, Tac31A, encoded on a high-copy-number plasmid. In the original characterization of the system, up to 88% of the β-galactosidase (β-Gal) activity of L. lactis KP1 was detected in the cell supernatant, with no discernible effect on cell growth, lysis, or membrane integrity (24). The leaky system, like the S. aureus autolysin-controlled system (38), is selective. While β-Gal (117 kDa) and tetanus toxin fragment C (47 kDa) were externalized from the leaky system, PepXP (176 kDa) and five other peptidases (30 to 100 kDa) were not (24, 40). None of the peptidases, PepA, PepC, PepN, PepO, and PepXP, have transmembrane helices or signal peptide sequences, suggesting that all are cytosolic. The determining factor in whether a protein is externalized is unknown. The protein masses of the tested proteins (as monomers) were evidently not a factor. Remarkably, in this work, virtually all of the cytoplasmic Cry5B (140 kDa) and tCry5B (79 kDa) proteins was externalized by the leaky system, with no effect on cell growth. Cell membrane permeability appears to be unaffected in log-phase leaky cells, but the leaky phenotype appears to cause considerable permeability in stationary-phase cells.

The Tac31A activator regulates a late phage promoter of a prophage in L. lactis KP1 (24). While L. lactis MG1363 carries prophage sequences, β-Gal was not externalized from this strain, suggesting that Tac31A is not an activator of a holin/lysin promoter for this strain (24, 41). Investigation of recombinant phages arising from KP1 has indicated that there are two distinct regions of the KP1 genome which can contribute to recombinant phages and therefore must contain prophage sequences (42). KP1 holin and lysin genes were not identified in the recombinant phages, which arose in response to a phage resistance mechanism which targeted phage replication. One of the temperate phages can be induced from KP1 by using mitomycin C, and it closely resembles temperate phages of two other lysogenic L. lactis strains (43). Activation of the late prophage transcript(s) is weak, allowing the cells to continue to grow (24). Further investigation will be required to determine the mechanism of cytosolic protein release by the leaky Lactococcus system.

*In vivo* challenges to C. elegans worms with cell lysates of L. lactis expressing Cry5B and tCry5B demonstrated biological activity and abated worm development. Nisin-induced expression of both Cry toxins showed distinct biological activity, in synergism with or in addition to the effect of nisin, whereas only Cry5B showed activity expressed from the base constitutive expression plasmid, pTRK593.

We have shown here that active full-length and truncated Bt Cry5B and tCry5B proteins can be expressed in L. lactis cells, both constitutively and through nisin induction. Both forms of Cry5B are found intracellularly and in culture supernatants from log-phase L. lactis MG1363 and KP1 cells when expressed constitutively. The externalization of Cry5B occurs without any detectable disruption in cell growth or membrane integrity and in the absence of increased cell death. After nisin induction of log-phase L. lactis MG1363, increasing amounts of Cry5B are detected intracellularly with increasing concentrations of nisin. However, Cry5B is not detected in the cell supernatants, perhaps because the 3-h assay is not long enough to allow for externalization. When Cry5B is expressed constitutively in L. lactis KP1 by use of the leaky lactococcus system, virtually all of the protein is found in the cell supernatants, and none is detected intracellularly. Together, these systems provide multiple options for expression of active Cry5B and tCry5B proteins from L. lactis for possible use as oral anthelminthics.
